# Altering HIF-1*α* Through 2,3,7,8-Tetrachlorodibenzo-*p*-Dioxin (TCDD) Exposure Affects Coronary Vessel Development

**DOI:** 10.1007/s12012-012-9194-7

**Published:** 2012-12-24

**Authors:** Jamie Wikenheiser, Ganga Karunamuni, Eddie Sloter, Mary K. Walker, Debashish Roy, David L. Wilson, Michiko Watanabe

**Affiliations:** 1 Department of Anatomy and Neurobiology, University of California, Irvine School of Medicine, 1001 Health Sciences Rd, 306D Med Surg II, Irvine, CA 92697 USA; 2 Department of Pediatrics, Rainbow Babies and Children’s Hospital, Case Western Reserve University, School of Medicine, 2101 Adelbert Road, Cleveland, OH 44106-6011 USA; 3 WIL Research, 1407 George Rd, Ashland, OH 44805 USA; 4 Department of Pharmaceutical Sciences, College of Pharmacy, University of New Mexico, 2502 Marble NE, Albuquerque, NM 87131 USA; 5 BioInvision Inc, 781 Beta Dr. Ste E, Cleveland, OH 44143 USA

**Keywords:** Cardiovascular development, TCDD, Hypoxia-inducible factor, AhR, Coronary anomalies, Hypoxia

## Abstract

Differential tissue hypoxia drives normal cardiogenic events including coronary vessel development. This requirement renders cardiogenic processes potentially susceptible to teratogens that activate a transcriptional pathway that intersects with the hypoxia-inducible factor (HIF-1) pathway. The potent toxin 2,3,7,8-Tetrachlorodibenzo-*p*-dioxin (TCDD) is known to cause cardiovascular defects by way of reduced myocardial hypoxia, inhibition of angiogenic stimuli, and alterations in responsiveness of endothelial cells to those stimuli. Our working hypothesis is that HIF-1 levels and thus HIF-1 signaling in the developing myocardium will be reduced by TCDD treatment in vivo during a critical stage and in particularly sensitive sites during heart morphogenesis. This inadequate HIF-1 signaling will subsequently result in outflow tract (OFT) and coronary vasculature defects. Our current data using the chicken embryo model showed a marked decrease in the intensity of immunostaining for HIF-1*α* nuclear expression in the OFT myocardium of TCDD-treated embryos. This area at the base of the OFT is particularly hypoxic during normal development; where endothelial cells initially form a concentrated anastomosing network known as the peritruncal ring; and where the left and right coronary arteries eventually connect to the aortic lumen. Consistent with this finding, anomalies of the proximal coronaries were detected after TCDD treatment and HIF-1*α* protein levels decreased in a TCDD dose-dependent manner.

## Introduction

The contaminant 2,3,7,8-Tetrachlorodibenzo-*p*-dioxin (TCDD) is a halogenated aromatic hydrocarbon that resists chemical and biological degradation, thereby persisting in the environment and increasing the chances of human exposure. TCDD is highly lipophilic, which promotes its bioaccumulation in the food chain, leading to humans accumulating these chemicals over a lifetime via a diet of meat, poultry, and dairy products, mother’s milk, and to a lesser extent exposure from inhalation of smog and cigarette smoke [1, 2]. Among the various adverse effects caused by TCDD in animal experiments, its carcinogenic effects have caused particular concern. TCDD does not behave like a “complete carcinogen,” that is, no DNA binding of the parent compound or metabolites could be detected. However, enhanced oxidative damage of hepatic DNA was observed, probably resulting from a dramatic induction of cytochrome P450 enzymes, which are under the regulatory, transcriptional control of the TCDD-activated aryl hydrocarbon receptor [2]. The chick embryo can be utilized as a highly sensitive model to help elucidate the mechanism underlying TCDD-induced cardiovascular toxicity during development because under low levels of TCDD, it can still produce edema, hemorrhage, and embryonic mortality [3, 25].

**Fig. 1:**
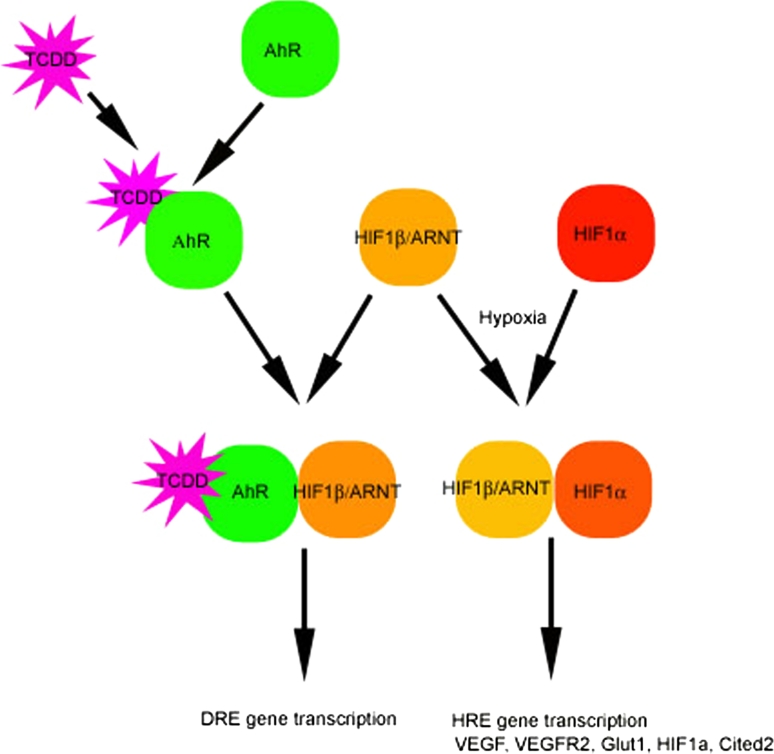
Proposed intersection of the toxin and hypoxic pathway. Both pathways require HIF-1*β*/ARNT

Studies using animal models provide evidence that the vasculature is a target of TCDD and related chemicals, and that toxicity is mediated by the activation of the aryl hydrocarbon receptor (AhR). The AhR is a cytosolic, ligand-activated transcription factor that belongs to the basic helix-loop-helix-PAS (bHLH-PAS) superfamily of DNA-binding proteins. TCDD binds the AhR with high affinity and stimulates its translocation into the nucleus, where it dimerizes with another bHLH-PAS protein, ARNT (or HIF-1*β*). Although no specific genes involved in angiogenesis or vasculogenesis have been identified to be transcriptionally regulated by the AhR/ARNT complex, other studies have suggested that sustained activation of the AhR leads to changes in the expression of known angiogenic stimuli [1]. TCDD inhibition of coronary development is preceded by a decrease in myocyte proliferation and an increase in cardiac apoptosis [4]; exogenous VEGF rescues the inhibitory effect TCDD has on vasculogenesis [5]; TCDD exposure reduces myocardial hypoxia and VEGF expression [6] and also reduces endothelial cell responsiveness to angiogenic stimuli [7]. These results are consistent with the hypothesis that TCDD-activated AhR may compete with the hypoxia-inducible factors for binding to the ARNT protein (Fig. 1).

HIF-1*α* expression levels have recently been shown to play a role in not only the normal development of coronary vasculature but also the development of potentially clinically relevant coronary artery anomalies [8, 9]. The aim of this study was to find evidence that TCDD might be interfering with HIF-1-mediated transcriptional regulation. Our working hypothesis is that HIF-1*α* levels and thus HIF-1 signaling in the developing myocardium will be reduced by TCDD treatment in vivo during critical stages in chick heart morphogenesis and coronary vessel development. This inadequate HIF signaling will in turn lead to the misexpression of HIFs and abnormal coronary vasculature development.

## Materials and Methods

### Preparation and Injection of Chicken Embryos

Plymouth Rock, Barred variety, or White Leghorn (*Gallus gallus*) fertile chicken eggs (Privett Hatchery, Portales, NM and Charles River-Avian Vaccine Services, CT) were first weighed to determine the average and range of weight for the preparation of the dosing solutions and then incubated at 37.5 °C at 55 % humidity in a forced-draft JamesWay incubator under normoxic conditions (20.8 %) until the embryos reached appropriate stages for each experiment (HH 30 & 35) [10]. The eggs were placed on an automatic rotator such that they were rocked back-and-forth approximately once per every 2 h. Eggs were incubated until day 4.5 (Stage 25) when they were removed from the incubator and laid horizontally. The blunt end was wiped with ethanol and a hole made in the shell into the aircell with a sharp probe. The dosing solution (vehicle control—corn oil, TCDD dissolved in corn oil) was administered with a 100-μl gas tight Hamilton syringe with a 26-g beveled removable needle. The needle was inserted into the hole in the eggshell to ~1 cm and 10 μl of the dosing solution was dispensed into the aircell. The egg was placed in a vertical position with the blunt end upwards for further incubation. Dosings were with the vehicle control of corn oil alone and 1.0 pmol and 3.0 pmol of TCDD/gm in 10 μl of corn oil. Stages of experimental exposure were selected based on the results from a previous publication to target stages of active coronary vasculogenesis [9].

### Immunohistochemistry

Embryos were fixed in fresh 4 % paraformaldehyde at 4 °C for 1 h, prepared for cryosection through a series of sucrose solutions, frozen and cross-sectioned in either the frontal or transverse plane. The 10–12-μm thick sections were collected on treated (Plus, Fisher) slides and immunostained. The primary antibodies anti-HIF-1*α* (Gift of Dr. Faton Agani) and anti-*α*-smooth muscle actin conjugated with Cy3 (Sigma, St Louis, MO) were incubated overnight at 4 °C at a dilution of 1:500 and 1:400, respectively. Anti-HIF-1*α* was detected with goat anti-rabbit IgG antibody, conjugated with biotin (Vector, Burlingame, CA) at 1:200 dilution, and the signal was amplified with the TSA system with fluorescent tyramide signal (PerkinElmer, Boston, MA) per manufacturers instructions. Stained sections were observed with an inverted fluorescence microscope (Nikon Diaphot 200, Japan) or stereomicroscope (Leica MZ16F, Leica Microsystems, Wetzlar, Germany) and images were captured with a Q-Imaging Retiga EXi FAST 1394 digital camera and Q-capture software (Q-Imaging Burnaby, BC, Canada). Digital images were adjusted with Adobe Photoshop CS5 software. Images of the negative controls were captured with the same exposure as those of the experimental samples at the same magnification and adjusted in parallel.

### Western Blots

Stage 30 embryonic chicken whole hearts were homogenized with a sonicator under ice within an ice-cold lysis buffer [50 mM Tris-HCl (pH 7.4), 150 mM NaCl, 1 % NP-40, 1 % Triton X-100, 0.25 % Na-deoxycholate, 0.1 % SDS, 1 mM EDTA, and a protease inhibitor cocktail (Complete Mini, EDTA-free tablets (Roche, Mannheim, Germany)] and stored in a −80 °C freezer until needed. Protein concentration was determined by using the DC protein assay (Bio-Rad Laboratories Inc, Hercules, CA). A total of 100 μg (50 mg for Tie2) of the whole heart protein lysate was electrophoresed on an 8 % SDS–PAGE gel and then transferred onto a polyvinylidenedifluoride (PVDF) membrane (Millipore, Bedford, MA). Membranes were blocked for 1 h at room temperature with 5 % nonfat milk in Tris-buffered saline with 1 % Tween (TBST). The primary antibody rabbit polyclonal anti-HIF-1*α* (Gift of Dr. Faton Agani) and rabbit polyclonal anti-Tie2 (Santa Cruz Biotechnology, Inc) were incubated at a dilution of 1:500 and 1:1,000, respectively, overnight at 4 °C. The monoclonal antibody anti-*β*-tubulin was used for the loading control (1:100,000). After washing, the blots were incubated with an anti-rabbit IgG (H&L) HRP-linked antibody (Cell Signaling, Beverly, MA) for 1 h at room temperature at a dilution of 1:5,000 (1:15,000 Tie2). Anti-mouse IgG (H&L) HRP-linked antibody (Cell Signaling, Beverly, MA) was used against *β*-tubulin at a dilution of 1:100,000 for 1 h at room temperature. Signals were detected using an enhanced chemiluminescence detection system (ECL) (Pierce Chemical Co., Rockford, IL).

## Results

**Fig. 2:**
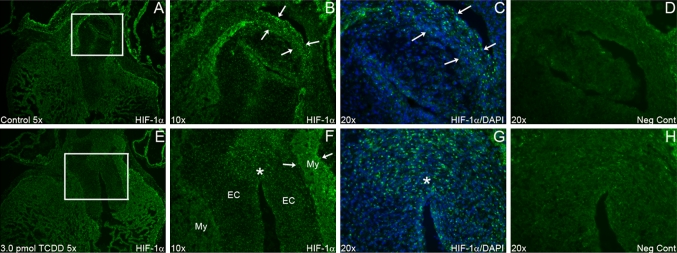
HIF-1α staining of control verses TCDD-treated embryos. Specific sites of immunofluorescent staining for HIF-1*α* near the OFT myocardium of a stage 30 chicken embryonic heart. HIF-1*α* nuclear-localized immunostaining intensity and frequency was high in the control (**a**–**c**) OFT myocardium (*arrows* *in* **b**, **c**) while lower in the TCDD-treated embryos (**f**–**h**). However, TCDD-treated embryos had increased HIF-1*α* staining within the endocardial cushions (**g** *between arrows*, **h**). *My* myocardium and *EC* endocardial cushions. Bright staining in **d** and **h** is autofluorescence of red blood cells and not that of nuclear-localized HIF-1*α* staining

**Fig. 3:**
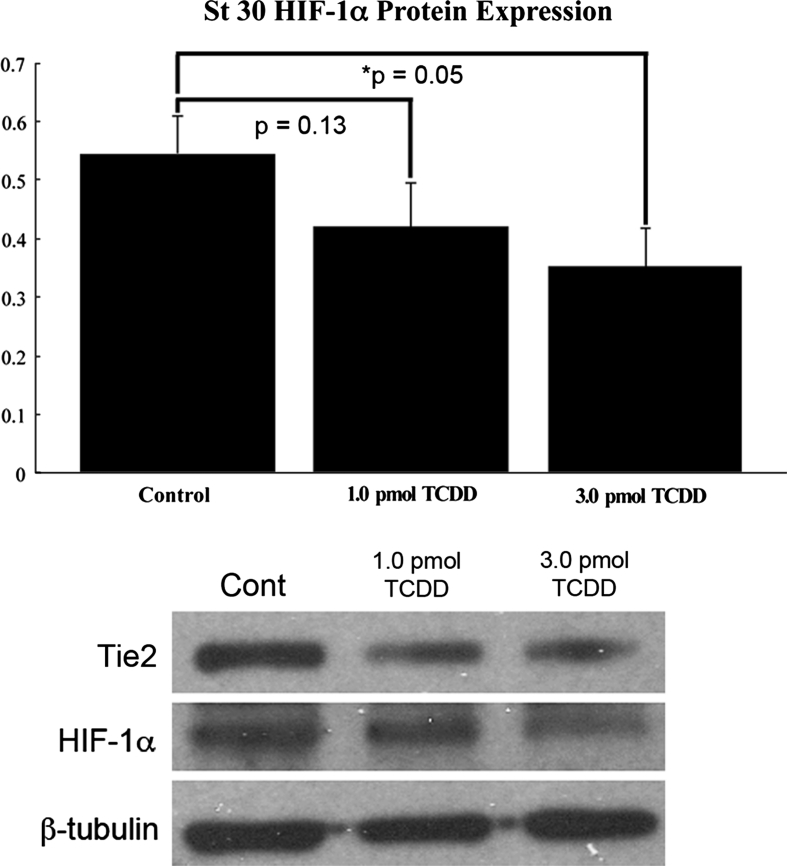
HIF-1*α* and Tie2 protein expression after TCDD exposure. Protein expression as qualitatively assessed by Western blot decreased for both HIF-1*α* and Tie2 after exposure to 1.0 pmol and 3.0 pmol of TCDD. Decreases in HIF-1*α* were dose-dependent and were not statistically significant until embryos were exposed to 3.0 pmol of TCDD

The hypothesis tested in this study is that TCDD may act to alter coronary vessel development by reducing HIF-1*α* nuclear localization and thus HIF-1 signaling in the developing myocardium. HIF-1*α* nuclear-localized staining was reduced markedly in most areas of the heart but most notably in the stage 30 (ED 6.5) OFT myocardium under 3.0 pmol of TCDD (Fig. 2). These reductions within the OFT myocardium were in the region where the left and right coronary arteries will eventually attach to the aorta (stage 32/ED 7.5). We also noted that there was an increased level of positive HIF-1*α* nuclear-localized immunostaining within the endocardial cushions near the OFT of the stage 30 embryo. HIF-1*α* protein levels of whole heart extracts at stage 30 qualitatively assessed by Western blot analysis decreased. This decrease in HIF-1*α* protein expression was TCDD dose-dependent. Quantitative analysis of the Western blot analyses indicated that the dose of 3.0 pmol but not 1.0 pmol of TCDD resulted in a significant decrease in HIF-1*α* protein expression (Fig. 3). We also found that the expression of the Tie2 protein at stage 30 was decreased in a TCDD dose-dependent manner (Fig. 3). Tie2 is important for the regulation of endothelial cell differentiation during vascular remodeling and maturation and although not a direct target of HIF-1*α* is an indicator of vascular differentiation [11, 12].

Embryos were then exposed to a vehicle control, 1.0 or 3.0 pmol of TCDD/gm and incubated until stage 35 (ED 9) to assay the anatomy of the proximal segments of the coronary arteries. All but two embryos (2 out of 12) survived the vehicle control injections and these embryos displayed no gross abnormalities when examined stereoscopically prior to dissection. All control embryos analyzed in histological section (*n* = 10) displayed normal anatomy, with neither coronary artery anomalies nor outflow tract septal defects. A total of 7 of 12 embryos survived to stage 35 after exposure to 1.0 pmol of TCDD/g. These TCDD-treated embryos exhibited no gross abnormalities as viewed intact under the stereoscope. They appeared similar in size and morphology with no noticeable defects similar to the 10 surviving control injected embryos. However, after transverse sections were stained for *α*-smooth muscle actin and coronary arteries were examined, 1 of 7 embryos displayed a coronary defect. The defect was a retro-aortic branch off the left coronary artery that wrapped around the aorta and inserted near the posterior cusp where there is normally no coronary artery stem. This same embryo had a septal defect of the OFT.

**Fig. 4:**
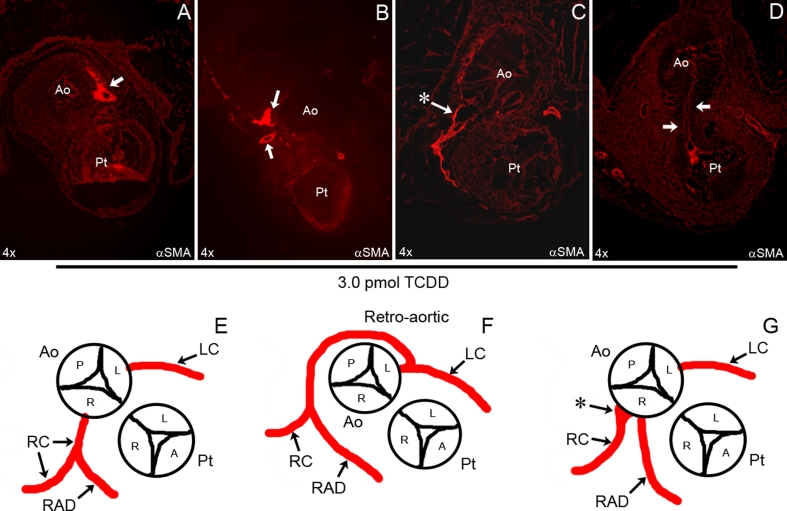
Anatomy of the proximal coronaries after TCDD exposure. Cryosections of hearts were immunostained with anti-smooth muscle actin to identify coronary arteries. The pattern of the proximal coronary arteries changed and septal defects were seen after a 3.0-pmol exposure to TCDD. Coronary arteries and OFT septal defects were observed in 1 of 7 embryos after a 1.0-pmol exposure to TCDD (data not shown). After treatment with 3.0 pmol of TCDD, 5 of 8 embryos displayed coronary artery anomalies. The anomalies included retro-aortic coronary (**a**, **f**), double right coronary (**b**, **g**), or an incomplete lumen (**c**). 4 of 8 embryos displayed septal defects (*arrows in* **d**) after exposure to 3.0 pmol TCDD. Diagrams of the proximal attachments of the left and right coronary arteries in a stage 35 (ED 9) embryo were deduced from the observation of serial transverse sections (**e**–**g**). *Asterisks* in **c** and **g** represent a sinus or ampulla near the attachment of the right coronary artery to the aorta. *A* Anterior, *P* posterior, *L* left, *R* right, *Ao* aorta, *Pt* pulmonary trunk, *LC* left coronary, *RC* right coronary, *RAD* right anterior descending—present in the chicken

After injection of 3.0 pmol of TCDD/g, only 8 of 15 embryos survived to stage 35. Compared to control embryos (*n* = 10), 3.0 pmol/g injected embryos had increased mortality, hemorrhaging, and a failure of the body cavities to close. Examination of the sections revealed coronary anomalies (5/8) and OFT septal defects (4/8) in these treated embryos. All of the embryos had some form of cardiac defect with 2 of 8 having both coronary artery and septal defects of the OFT. There was a coronary artery anomaly produced after a 3.0 pmol/g exposure that was similar to an anomaly seen after a 1.0 pmol/g exposure. Many of the coronary anomalies observed were of the retro-aortic type in which a coronary, usually a branch off the left coronary artery, inserted back near the posterior cusp of the aorta. There were also multiple embryos that displayed double right coronary arteries (Fig. 4; Table 1). In one case, there was a dilation of the right coronary vessel at the point of connection with the lumen of the aorta. The flat and irregular morphology of these anomalous vessels resembled venous vessels rather than arteries.

**Table 1:** A Summary of dosage and mortality rates. B Summary of septal and coronary artery anomalies

Treatment	Injected	Collected	Dead	Description
*A*				
Vehicle	12	10 (83 %)	2 (17 %)	Normal
1.0 pmol of TCDD/g	12	7 (58 %)	5 (42 %)	Similar to controls
3.0 pmol of TCDD/g	15	8 (53 %)	7 (47 %)	Hemorrhaging and body cavities failed to close

Treatment	Septal defects	Coronary anomalies	Description
*B*			
Vehicle	0/10 (0 %)	0 (0 %)	Normal
1.0 pmol of TCDD/g	1/7 (14 %)	1/7 (14 %)	Most are normal; retro-aortic coronary anomaly was observed in one embryo along with a septal defect
3.0 pmol of TCDD/g	4/8 (50 %)	5/8 (63 %)	All but one embryo was abnormal; retro-aortic and double right coronary anomalies; flattened lumens; 2/8 embryos had both septal and coronary defects

## Discussion

TCDD treatment reduced the area of cells in the OFT myocardium that was positive for nuclear-localized HIF-1*α* as well as the overall level of HIF-1*α*. TCDD also caused coronary anomalies that resembled those induced in a previous study by altering the environmental level of oxygen or by directly altering HIF-1*α* expression levels using an engineered adenovirus vector [9]. These results were consistent with our hypothesis (Fig. 1) that the TCDD-activated AhR was competing with HIF-1*α* for ARNT and was therefore preventing or displacing HIF-1*α* from forming a stable heterodimer that could accumulate in the nucleus with the resulting consequence that downstream vasculogenesis and angiogenesis factors failed to regulate normal coronary vessel patterning. TCDD thus may have altered the hypoxic template mediated by HIF-1. The major difference between this study and a previous one [9] was that in this study, HIF-1*α* levels were reduced resulting in coronary artery anomalies as well as septal defects. The previous study showed increases in HIF-1*α* expression under adenovirus and hypoxic/hyperoxic conditions and resulted in coronary artery anomalies as well. The coronary anomalies in both studies displayed similar patterns. This supports that deviation of proper HIF-1*α* expression either up or down will result in coronary artery anomalies.

Nascent coronary vessels begin to form at stage 27 and attach to the systemic circulation by stage 32 [13]. A capillary-like network (peritruncal ring) surrounds the base of the outflow tract and select portions penetrate the aorta, recruits smooth muscle cells, and emerges as two main coronary artery stems connecting to the aorta [14, 15]. The proximal segment of the coronary arteries develops by endothelial ingrowth from the peritruncal ring rather than by endothelial outgrowth from the aorta [14–16] and is invariably located at the same locations relative to the aortic leaflets. The mechanism that results in this stereotyped pattern of the proximal coronary arteries is unknown. We previously showed that there is differential microenvironmental hypoxia in the developing heart creating a “hypoxic template” that precedes coronary vessel development [8] and that HIF-1*α* regulation plays an important part in the establishment of the proximal coronary artery patterns [9]. With the addition of our TCDD data, there is now another example correlating the disturbance of HIF-1*α* nuclear expression and abnormal patterns of the proximal coronary arteries.

In addition to coronary anomalies, TCDD also induced outflow tract septal defects (Fig. 4) that we did not observe with other methods of altering HIF-1*α* expression [9]. However, OFT abnormalities have been observed when embryos were exposed to conditions that more severely increased HIF-1*α* levels in the myocardium [17, 18]. These abnormalities while striking were not primarily OFT septal defects. We did note that HIF-1*α* levels within the mesenchymal cells of the outflow tract cushions were elevated after TCDD treatment that may account for the failure or delay of OFT septation. Thus, the outflow tract cushion cells may also be affected by TCDD. Zebrafish embryos exposed to TCDD are known to have abnormal outflow tract development associated with abnormal function [19].

TCDD created proximal coronary anomalies located at the base of the OFT and OFT septation defects. In view of the location of these defects, it is relevant to note that the gene expression of cytochrome P4501A4 that is induced by TCDD-activated AhR is particularly concentrated in the outflow tract myocardium after TCDD exposure [20, 21]. This region is also highly hypoxic as determined by hypoxia indicator EF5 and HIF-1*α* immunostaining [8, 17, 22]. Thus, this region of the heart may be particularly subject to the competition between AhR and HIF-1*α* for ARNT (Fig. 1).

Our finding that vasculogenesis protein Tie2 levels decreased with TCDD exposure (Fig. 3) is consistent with findings from a recent report. In a study using the rat animal model, exposure to TCDD decreased the size of maternal blood sinusoids, caused constriction of fetal capillaries in the placenta, and a decreased expression level of Tie2 mRNA [23]. Tie2 is an endothelial receptor tyrosine kinase which is highly expressed in endothelial cells and its interaction with protein growth factors known as angiopoietins shows that they play a crucial role in remodeling and maturation of embryonic vasculature [24]. We used Tie2 as an indicator of vascular differentiation in a previous study where we discovered that Tie2 protein levels in embryo heart extracts increased significantly along with HIF-1*α* levels under both hypoxic and hyperoxic conditions [9]. In the current study, we found the opposite effect, TCDD exposure reduced Tie2 protein levels along with HIF-1*α* levels thus supporting that differentiation of coronary vessels was compromised.

Our findings support that coronary vessel anomalies may result from TCDD exposure. While the TCDD-induced anomalies in the avian model may have no impact prior to hatching, we speculate that those that created sharp angles and dilatations in the vessels may increase the risk of ischemic heart disease, atherosclerosis, or coronary aneurysms in later life.

TCDD exposure induced both coronary artery anomalies and reduced HIF-1a protein expression. These results are consistent with the hypothesis that competition between the TCDD-activated AhR and HIF-1a for their heterodimer partner ARNT (Fig. 1). Further mechanistic studies, perhaps following the consequences of a knockout or knockdown of the AhR, would be required to provide proof for this hypothesis.
